# The orientation and stability of the GPCR-Arrestin complex in a lipid bilayer

**DOI:** 10.1038/s41598-017-17243-y

**Published:** 2017-12-05

**Authors:** Dali Wang, Hua Yu, Xiangdong Liu, Jianqiang Liu, Chen Song

**Affiliations:** 1 School of Physics, Shandong University, Jinan, 250100 China; 2 Center for Quantitative Biology, Academy for Advanced Interdisciplinary Studies, Peking University, Beijing, 100871 China; 3 Peking-Tsinghua Center for Life Sciences, Academy for Advanced Interdisciplinary Studies, Peking University, Beijing, 100871 China

## Abstract

G protein-coupled receptors (GPCRs) constitute a large family of membrane proteins that plays a key role in transmembrane signal transduction and draw wide attention since it was discovered. Arrestin is a small family of proteins which can bind to GPCRs, block G protein interactions and redirect signaling to G-protein-independent pathways. The detailed mechanism of how arrestin interacts with GPCR remains elusive. Here, we conducted molecular dynamics simulations with coarse-grained (CG) and all-atom (AA) models to study the complex structure formed by arrestin and rhodopsin, a prototypical GPCR, in a POPC bilayer. Our results indicate that the formation of the complex has a significant impact on arrestin which is tightly anchored onto the bilayer surface, while has a minor effect on the orientation of rhodopsin in the lipid bilayer. The formation of the complex induces an internal change of conformation and flexibility in both rhodopsin and arrestin, mainly at the binding interface. Further investigation on the interaction interface identified the hydrogen bond network, especially the long-lived hydrogen bonds, and the key residues at the contact interface, which are responsible for stabilizing the complex. These results help us to better understand how rhodopsin interacts with arrestin on membranes, and thereby shed lights on arrestin-mediated signal transduction through GPCRs.

## Introduction

Signal transduction is a fundamental biological process in organisms. Membrane protein at the cell interface serves as the medium between cell’s external and internal environments^1^. The most enormous membrane protein family is G protein-coupled receptor (GPCR) which contains hundreds of membrane-embedded proteins. According to amino acid sequence similarity, GPCRs can be classified into six families: A (rhodopsin), B (secretin), C (metabotropic glutamate), D (Fungal mating pheromone receptor), E (cAMP receptor), and F (frizzled). Among the six families of GPCR, rhodopsin-like receptors constitute the major group. These receptors are widely distributed in organisms, involving widespread protein families such as hormones and light receptors, and thus have attracted much attention^2,3^.

In the transmembrane signal pathway, GPCR can be mediated by the G proteins, the G-protein-coupled receptor kinases (GRKs), and the arrestins^4,5^. These three families of proteins affect different stages of signaling. When agonist stimulates the cell, G protein binds to GPCR and transport this stimulus to second-messenger-generating effectors. According to Violin *et al*.’s research, different agonists would activate different downstream responses^6^, which provides a new scope for GPCR-targeted drug discovery^7^. Herenbrink *et al*. recently revealed that the ligand-binding kinetics and temporal pattern of signal will influence agonists biased binding receptors^8^. After stimulations, GPCR requires desensitization to the stimulus and then returns to the membrane or degrades in lysosome. GRKs is able to phosphorylate the residues at C terminus of GPCR, which is a vital mechanism of receptor desensitization^9^. The phosphorylated GPCR then recruits β-arrestin to membrane and binds with it. This process makes G-protein unable to rebind with GPCR and finally leads to GPCR’s further desensitization^10^. Furthermore, β-arrestin mediates GPCR internalization and establishes contact between the receptor and clathin^11,12^. Then, GPCR will be degraded by lysosome or sent back to membrane. Meanwhile, β-arrestin reverts to the inactive state in the cytoplasm.

Arrestins are a small protein family compared to GPCR. It includes four subtypes: arrestin 1, arrestin 4, β-arrestin-1 (arrestin 2), and β-arrestin-2 (arrestin 3)^13^. Arrestins are located in intracellular compartment of unstimulated cell. β-arrestin can not only regulate desensitization of GPCR but also interact with numerous transcription factors such as JNK, ERK, and DGK to mediate signal transduction independently^14–19^. Some arrestins also have the function to connect GPCR to important downstream effectors of transduction signal just like G-protein in G-protein-dependent pathway. Some agonists are identified to preferentially activate either G-protein-dependent signal pathway or arrestin-dependent signal pathway^15^, which is called biased signaling^20^. Previous assays have suggested that GPCRs have different conformations between G-protein-dependent and β-arrestin-dependent transduction stage^21^, and the β-arrestin-biased GPCR conformation is similar to the ground state while that of G-protein-biased has more differences from the unexcited state^22^. Studying distinct receptor conformations will assist in developing new GPCR-targeted drugs with different biased pathways.

Despite considerable progress in understanding biological functions of GPCR and arrestin, their biophysical aspects of signaling across membrane remain to be understood, such as the mechanisms of activation of arrestin and how GPCR interacts with arrestin. Recently, a high-resolution X-ray structure of rhodopsin-arrestin complex has been published^23^. Lee’s study about this structure suggested that conformational rearrangement of arrestin provides key insights into the kinetics of receptor binding and arrestin activation^24^. Another research by Nuber *et al*. confirms conformational change when activated β-arrestin bind to receptors’ C terminus^25^. Lally *et al*. discovered the arrestin C-edge loops could hook the membrane like an anchor when arrestin binding to rhodopsin^26^. However, we still lack many details of the interaction interface between rhodopsin and arrestin, which are important for understanding this signal transduction pathway. In this paper, we conducted molecular dynamics simulations of the rhodopsin-arrestin complex in a POPC bilayer, rhodopsin alone in a POPC bilayer and arrestin alone near a POPC bilayer with all-atom (AA) and coarse-grained (CG) models. We analyzed their respective orientation to study the movement and degree of fluctuation of the complex and the orientation changes of rhodopsin and arrestin upon the complex formation. We discussed the feasibilities of AA and CG models in orientation analysis, analyzed the international conformation changes of rhodopsin and arrestin upon complex formation, and studied the interaction interface of the complex in detail, focusing on the long-lived hydrogen bonds and the key contact residues at the interface. Our results obtained by molecular dynamics simulation provide atomic information and help us to better understand the mechanism of how rhodopsin interacts with arrestin, and thereby shed further light on the signaling through the GPCR-Arrestin pathway.

## Methods

### Structure Preparation for Molecular Dynamics Simulation

The crystal structure^23^ of the rhodopsin-arrestin complex (PDB ID:4ZWJ) downloaded from the Protein Data Bank was used as our model system. Modeller^27,28^ was used to add and refine the three missing residues (residue IDs 340-342) which are located in the loop region of arrestin. The whole complex structure was used as the initial model for simulating the rhodopsin-arrestin complex system, and the separated rhodopsin and arrestin structures extracted from the complex model were used for simulating the standalone rhodopsin and arrestin respectively. We also downloaded several other rhodopsin (PDB IDs:2x72, 4a4m, 5dys, 2j4y) and arrestin structures (PDB IDs: 1g4m, 3ugu, 1ayr) with high sequence identity and calculated the root mean square deviation (RMSD) between each of them and the corresponding structure we used for MD simulations (more detailed results are shown in Supplementary Table S1 and Fig. S1). We constructed the system with the default protonation states and disulfide bonds as automatically recognized by *pdb2gmx* in Gromacs.

### Coarse-grained Molecular Dynamics Simulations

The structures extracted from the PDB (ID:4ZWJ) were used as the initial structures for coarse-grained (CG) simulations. The standalone rhodopsin, standalone arrestin, and the complex with and without elastic network (ELN) between rhodopsin and arrestin were put into a simulation box in which water and POPC molecules were placed randomly. After tens of nanoseconds free simulation, the POPC molecules would spontaneously assemble around rhodopsin to form a bilayer and arrestin would hook below the bilayer. Each system with the CG model was simulated for ten independent repeats. CG simulations were performed using the MARTINI 2.2 force field^29^. CG structures and topology files were generated with the script *martinize.py* ^29,30^. Before production molecular dynamics (MD) simulation we performed equilibrium procedure to relax the whole system. First the system was energy minimized (EM) for 2000 steps, and then 0.2 ns NVT equilibration was performed with a time step of 20 fs. V-rescale and LINCS (Linear Constraint Solver) was used for temperature coupling and bond constraints. After equilibration we ran ten 200 ns independent simulations with a time step of 20 fs. All molecular dynamics simulations were performed under the constant pressure ensemble (NPT). The V-rescale algorithm with coupling time 1.0 ps was used to maintain the temperature (310 K). Berendsen thermostat with coupling time 1.0 ps was used to maintain the pressure (1.0 bar). Long range electrostatics were calculated with the reaction-field method. The Van der Waals interaction was cut off at 1.1 nm.

### All-Atom Molecular Dynamics Simulations

**Figure 1:**
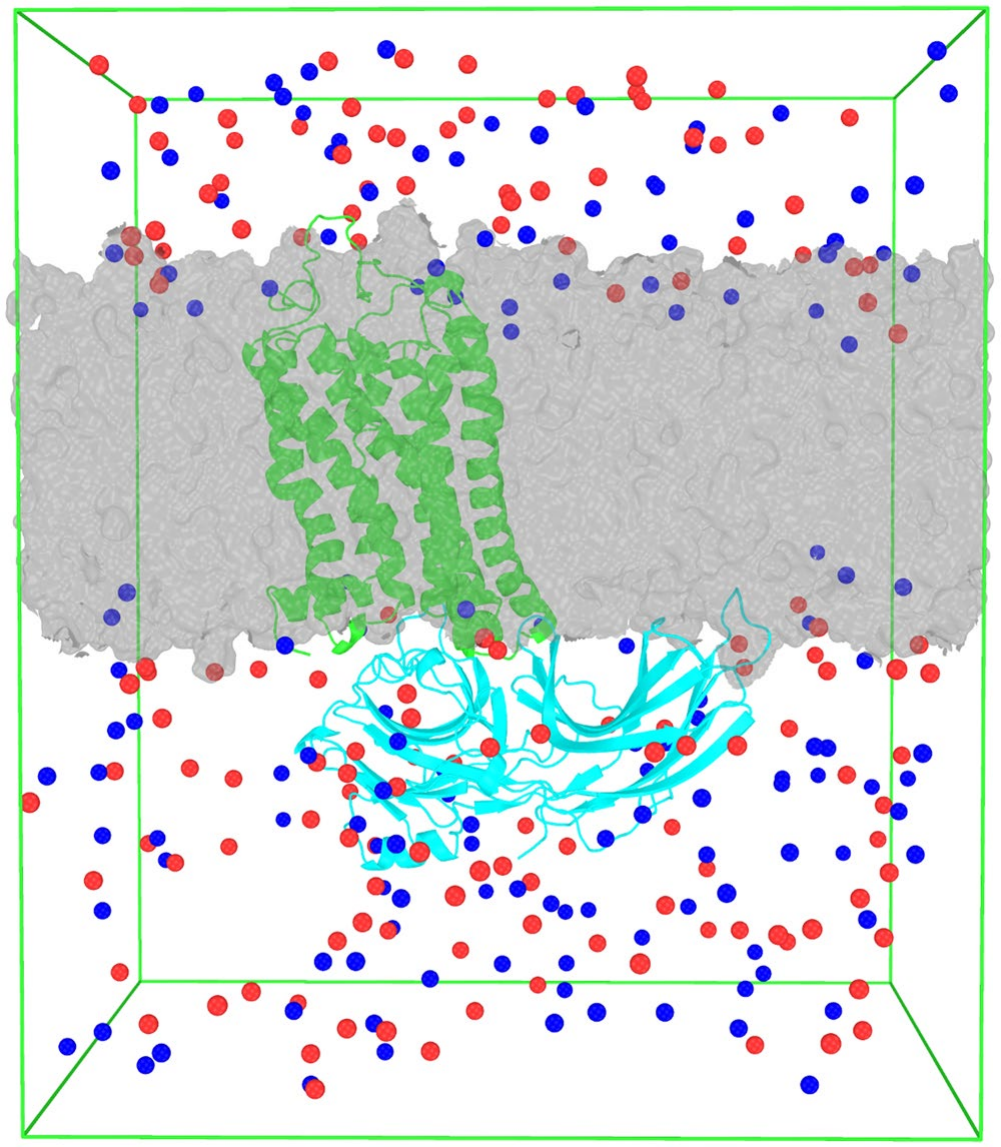
The initial system for molecular dynamics simulations. The gray surface represents the POPC lipid bilayer. The green cartoon indicates rhodopsin and the cyan cartoon indicates arrestin. The blue and red spheres represent Na^+^ and CL^−^ respectively. Water molecules were filled in the whole simulation box but are not shown here for clarity.

We used one of the final frames of the CG simulations as the template to build the all-atom model. The procedure involves two steps: 1) We used the *backward* tool^31^ to reverse the complex + POPC system obtained from CG simulations above to all-atom coordinates. 2) We aligned the complex crystal structure to all-atom structure generated by the backward method tool and replaced the *backward*-generated complex structure with the original crystal structure. We used *mdrun -membed* in Gromacs^32^ to remove the lipid and water molecules which overlap with the protein. The all-atom (AA) system was then used to perform molecular dynamics simulations with the AMBER99sb-ildn^33^ force field in Gromacs 5.0.2. The *Slipid* parameters of POPC^34,35^ developed by the Lyubartsev Group at Stockholm were incorporated into the AMBER99sb-ildn force field and used for our AA MD simulations. We added 145 Na^+^ and 146 Cl^−^ in a SPC/E^36^ water box to get a neutral system. The initial system is shown in Fig. 1. We equilibrated the system before the AA production MD simulations. We performed AA equilibration with 1000 kJ/mol·nm^2^ position restraint on all protein heavy atoms. After 5000 steps energy minimization, the system was equilibrated within NVT and NPT ensembles. We performed 0.5 ns NVT equilibration and 1.0 ns NPT equilibration with the time step of 2 fs. The long range electrostatic interactions were calculated using Particle-Mesh Ewald (PME). The reference temperature was 310 K. Semi-isotropic pressure coupling was used to maintain the pressure at 1.0 Bar. Neighborlists were updated every 10 steps and all-bonds were constrained with the LINCS algorithm. The Van der Waals cutoff was 1.0 nm. The Berendsen method was used for temperature coupling and pressure coupling in the equilibration. When the system was well-equilibrated at the desired temperature and pressure, we released the position restrains and ran 200 ns production MD simulations at NPT ensemble with V-rescale temperature coupling and Parrinello-Rahman pressure coupling.

### Data Analysis

**Figure 2:**
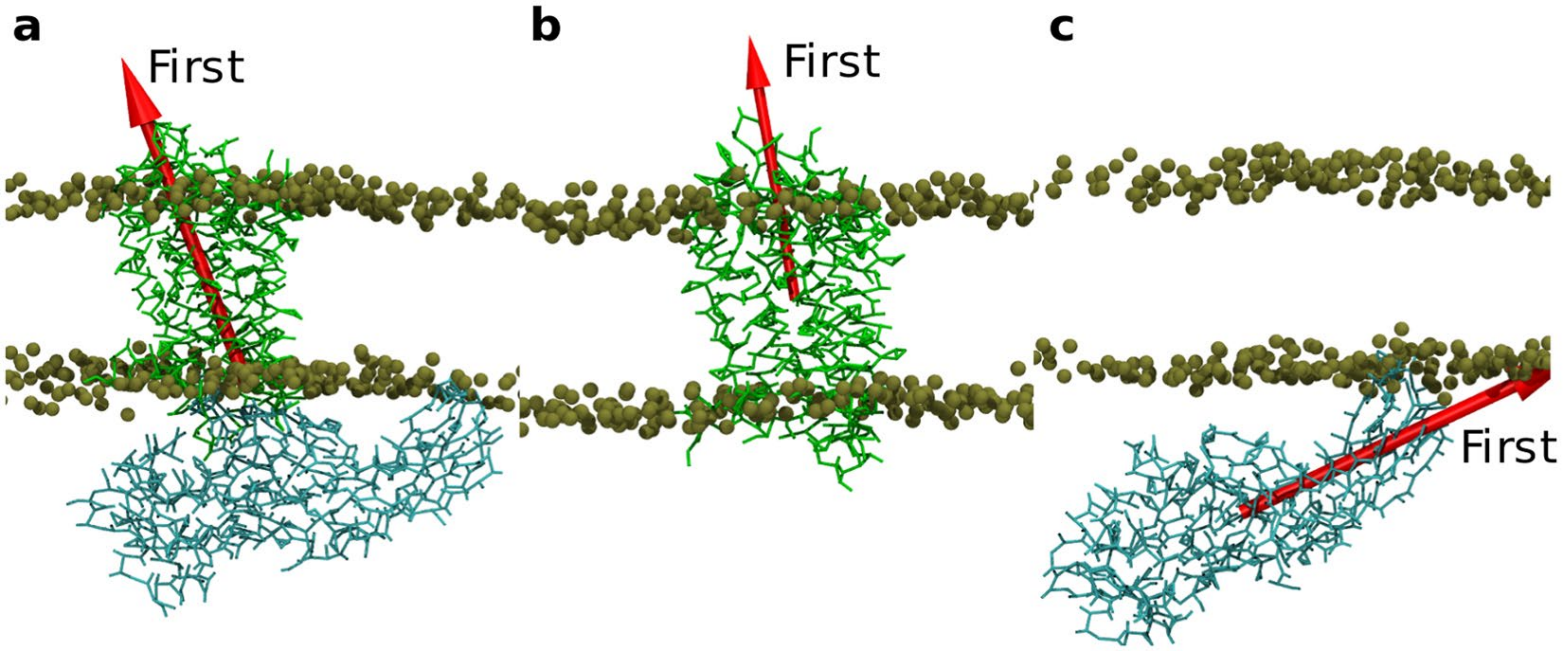
The first principal axis of the rhodopsin-arrestin complex, rhodopsin alone and arrestin alone. Red arrow represents the first principal axis. Brown spheres indicates the position of PO_4_ in the POPC bilayers (**a**) The first principal axis of the rhodopsin-arrestin complex in a POPC bilayer. (**b**) The first principal axis of the standalone rhodopsin, where rhodopsin was embedded into a POPC bilayer. (**c**) The first principal axis of the standalone arrestin near a POPC bilayer.

The MDAnalysis^37^ and Gromacs analysis tools were used to analyze the MD trajectories. For orientation analysis, we utilized *gmx -trjconv* in Gromacs to extract frames every 0.5 ns. Then we used MDAnalysis to calculate the first principal axis (Fig. 2) of each frame and calculated the angles of every principal axis with Z axis. For hydrogen bond analysis, the *hbond* tool of Gromacs was used to detect all the hydrogen bonds at the rhodopsin-arrestin interface. The “gmx mdmat” tool was used to analyze the contact residues at the rhodopsin-arrestin interface. For principal component analysis (PCA), the *covar* and *anaeig* tools of Gromacs were used to calculate the eigenvectors and project all the frames on the first and second eigenvectors.

## Results and Discussion

### Rhodopsin Embedded into Self-assembled Lipid Bilayers

In our coarse-grained self-assembly MD simulations, we found that the lipid molecules spontaneously form a bilayer around the rhodopsin within tens of nanoseconds, regardless of the presence or absence of arrestin. So by the end of the CG simulations, the rhodopsin was found to be embedded into a POPC bilayer spontaneously, and attached to arrestin which was fully exposed to water molecules when arrestin was present in the simulation system, as shown in Fig. 1.

### Orientation Analysis

#### The CG and AA Simulations Gave Converged Results Regarding the Protein Orientation in Lipid Bilayers

**Figure 3:**
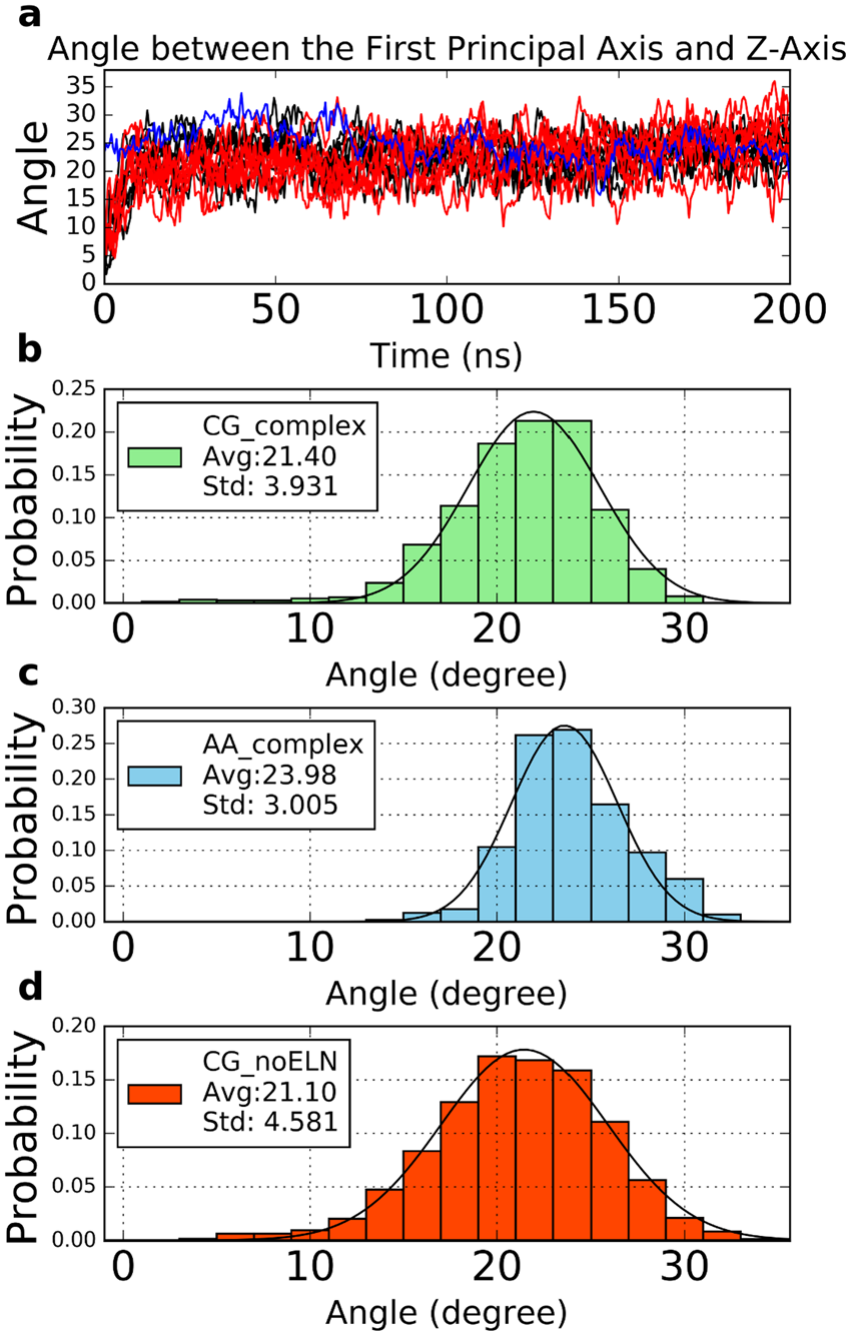
The orientation of the complex orientation in a POPC bilayer. The orientation of the complex is represented by the angle between the first principal axis of the rhodopsin-arrestin complex structure and the Z axis of the simulation box. (**a**) All the data from ten CG complex trajectories were plotted with black lines. Data from the ten CG complex without ELN were plotted with red lines. Blue line indicates the results from the AA MD simulation. (**b**–**d**) The angle probability distribution of the CG complex, AA complex and CG complex without ELN in MD, respectively.

To confirm whether CG model can replace AA model for orientation analysis, we calculated the angles between the first principal axis of the complex structure and the Z axis of the simulation box in the CG trajectories and AA trajectory. As shown in Fig. 3a, all of the first principal axes in these trajectories are unstable at the initial stage of the simulations, meaning the initial model was not the lowest-free-energy configuration. Gradually the principal axes became steady and the angles fluctuated in a small range, indicating the systems reached stable configurations during the simulation. From Fig. 3a, the tendency of these CG and AA simulations is consistent and the fluctuation ranges of the angles in the stable state are similar, which implies that CG simulations can work as well as AA simulations when analyzing the orientation of the protein in the trajectories. We further calculated the probability distribution of the complex orientation in CG and AA MD trajectories (Fig. 3b–d) as well as the average and standard deviation values. When comparing Fig. 3b and c we can see that the probability distributions of the protein orientation are similar in CG simulations and AA simulations, and the corresponding average and standard deviation values are very close. These results indicate that CG simulation is good enough for the orientation analysis. CG simulation can significantly improve calculation speed so that we can collect more trajectory data to get better convergence compared to AA model within the same computing time. Therefore, we will make our orientation analysis based on CG simulations below.

#### Elastic Network Has Little Effect on the Orientation of the Complex

Usually, we utilize an elastic network (ELN) to maintain the protein structure in CG MD simulations. To check whether this elastic network between rhodopsin and arrestin affects the orientation of the complex, we performed two sets of simulations: one with ELN within the whole structure, meaning there were ELNs between rhodopsin and arrestin, and the other without ELNs between rhodopsin and arrestin while still with ELNs within rhodopsin and arrestin respectively. Then we calculated the angles of the first principal axes of the complex with the Z axis for the two cases. As can be seen in Fig. 3a, the orientations of the complex are very similar in the trajectories with or without ELN between rhodopsin and arrestin after the complex finds its stable orientation. Comparing Fig. 3b and d, we can see the angle probability distributions of the CG complex and the CG complex without ELN are almost identical with each other. With ELN the relative motion of rhodopsin and arrestin is restrained by the elastic network, which should lead to less relative fluctuation of the complex compared with that without ELN. However, this restriction effect of ELN is trivial, and the arrestin without ELN can still bind to rhodopsin as tightly as with ELN, which implies that strong interactions exist between rhodopsin and arrestin and the application of ELN does not introduce any obvious artifacts.

#### The Formation of the Complex Has a Significant Influence on the Orientation of Arrestin while a Minor effect on that of Rhodopsin

**Figure 4:**
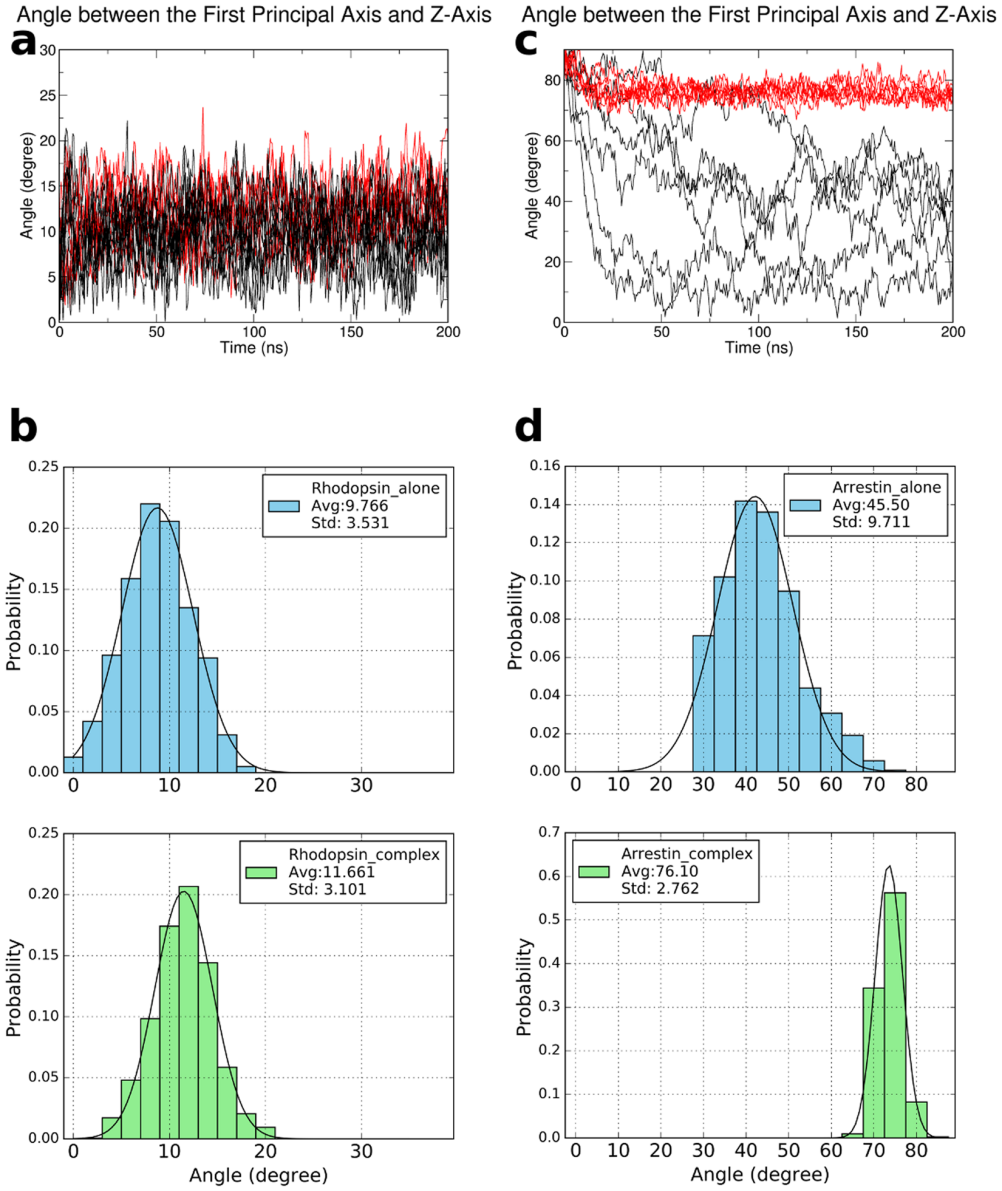
The orientation of rhodopsin and arrestin before and after the complex formation. All data from rhodopsin alone or arrestin alone were plotted with black lines, data from rhodopsin in complex or arrestin in complex were plotted with red lines. (**a**) The rhodopsin orientation change was subtle upon the formation of the complex. (**b**) The probability distribution of the angle between the first principal axis of rhodopsin and the Z-axis, before (top) and after (bottom) the complex formation. (**c**) The arrestin orientation was limited to a much smaller range upon the complex formation. (**d**) same as (**b**), but for arrestin.

It is interesting to compare the standalone rhodopsin structure and the standalone arrestin structure to the corresponding part in the complex to see whether the complex formation affected the orientation of the rhodopsin in membrane or that of arrestin near membrane. Figure 4a shows that the orientation angle of rhodopsin in the complex is almost the same as that of rhodopsin alone in the lipid bilayer. It seems that the complex formation makes no appreciable effect on the orientation of rhodopsin in a bilayer. The most probable angle shifts about 2° between rhodopsin in complex and rhodopsin alone (Fig. 4b), which is within the error bar. Rhodopsin exhibits very stable orientation before and after the complex formation, indicating that the complex formation does not have significant effect on rhodopsin’s orientation in a lipid bilayer, as can be seen from the small standard deviation values of the corresponding angle distribution. However for arrestin, we observed that the orientation angles of arrestin alone near a POPC bilayer in water differs a lot with that of arrestin in the complex. Also, the standalone arrestin shows broad distribution of orientation angle, in sharp contrast to arrestin in complex as can be seen from the angle distribution and standard deviation in Fig. 4c. This is distinct from rhodopsin’s behavior which is understandable as arrestin was immersed in water while rhodopsin was embedded in a lipid bilayer. Water molecule is much more fluid than lipid, therefore arrestin has more freedom and is more flexible before it binds to rhodopsin. In contrast, the orientation angles of arrestin in complex fluctuate to a much smaller range, around 70–80° degrees (Fig. 4d), indicating that the complex formation greatly limited the orientation of arrestin, which is consistent with Lally *et al*.’s work^26^. We should mention that, in two out of ten independent simulations, the arrestin misassembled into the bilayer, which we considered unrealistic and discarded for orientation analysis (more details are shown in Supplementary Fig. S2). Taken together, our data indicate that the complex formation has a significant influence on the orientation of arrestin, while only a minor effect on rhodopsin.

Usually CG simulations can give converging results about the membrane protein insertion and orientation in a lipid bilayer within 100 ns. To further validate the results have indeed converged within the 200 ns trajectories, we extended extra 2000 ns simulations for two of our CG simulations and analyzed the orientation in the extended trajectories. The results indicate that the orientation of the complex (Supplementary Fig. S3), the standalone rhodopsin or the standalone arrestin is indeed very stable in the extended simulations, indicating that the orientation of the proteins indeed converged within 100 ns in the CG simulations.

#### Internal Conformational Changes

**Figure 5:**
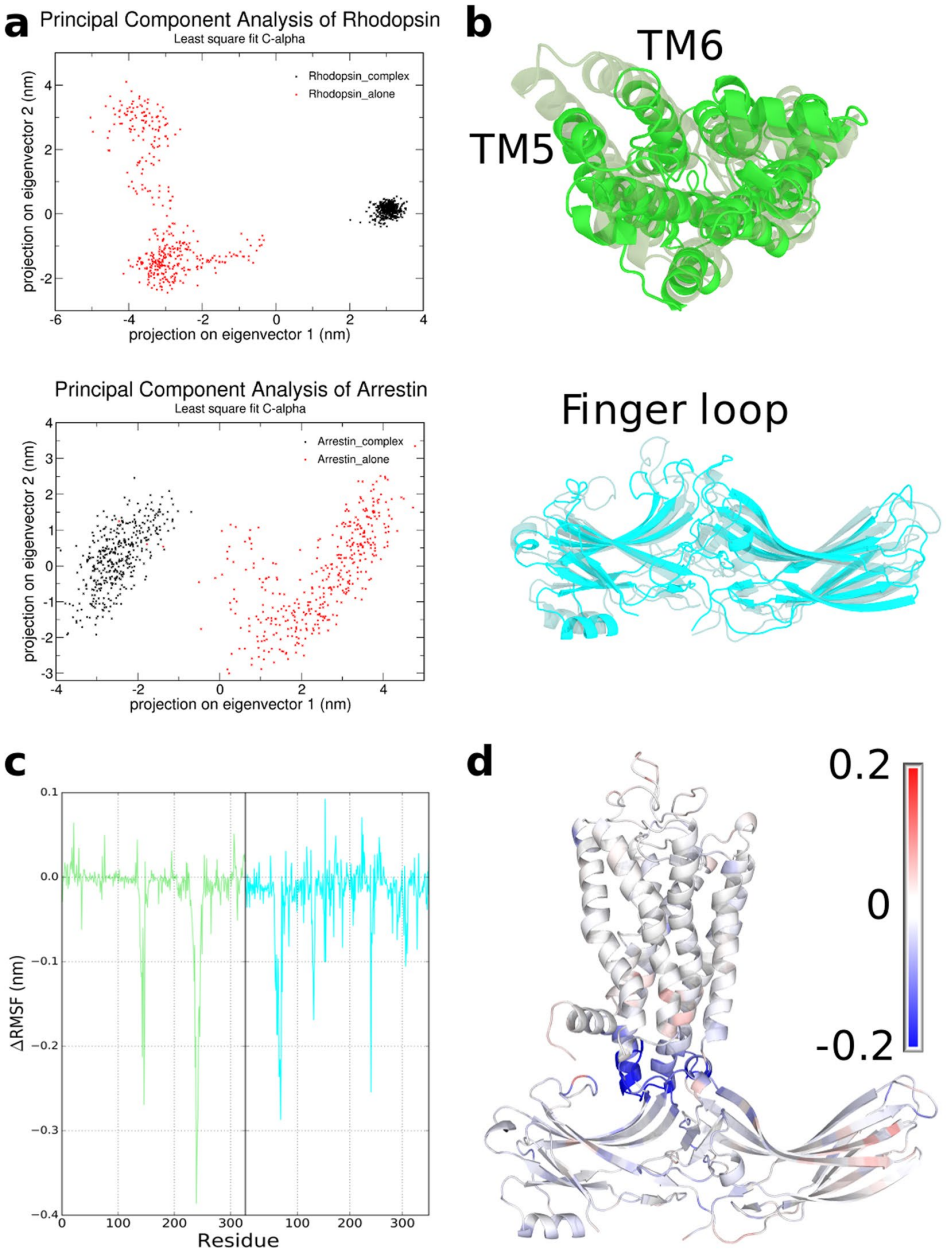
Principal component analysis (PCA) and RMSF change of rhodopsin and arrestin. (**a**) The results of PCA. (**b**) Two extreme conformations of rhodopsin and arrestin as determined by PCA. The transparent structure represents the conformation in complex, and the opaque structure represents the standalone conformation. (**c**) The RMSF change of the residues of rhodopsin and arrestin upon the complex formation. (**d**) The colored complex structure according to the RMSF change of the residues with the blue-white-red code.

In order to investigate the internal conformational changes of rhodopsin and arrestin upon the complex formation, we performed 200 ns AA MD simulations of the standalone rhodopsin and arrestin respectively. Then we did principal component analysis (PCA) with the standalone rhodopsin trajectory and the rhodopsin trajectory extracted from the complex simulation. The same analysis was done for arrestin. The results of PCA are shown in Fig. 5a. As can be seen, the data points from the standalone structures and their corresponding structures in the complex, colored in red and black respectively, are well separated, indicating that both rhodopsin and arrestin experienced appreciable internal conformational changes upon the complex formation. We extracted the two most different conformations (leftmost and rightmost points in the PCA plot) of rhodopsin and arrestin and overlay them in Fig. 5b. The top figure shows the intracellular view of rhodopsin, where we can see that TM5 and TM6 of rhodopsin in complex (transparent) had an outward movement compared to that in the standalone rhodopsin (opaque), which is the most significant internal conformational change of rhodopsin upon the complex formation. When arrestin was removed from the simulation system, the conformation of rhodopsin spontaneously changed back to the ground state with the TM5 and TM6 closely packed to the other helices (opaque). The bottom panel of Fig. 5b shows that the uppermost finger loop of arrestin moved towards rhodopsin upon the complex formation (transparent), which highlights the key role of this finger loop in rhodopsin-arrestin binding. The standalone arrestin (opaque) exhibits more similarities to the inactive state during our MD simulation.

### Rhodopsin-Arrestin Interface

#### Root Mean Square Fluctuation Analysis

What happens at the rhodopsin-arrestin interface is a fascinating question which may uncover the keys to understand the formation and stability of the complex. Since the complex is very stable and its orientation fluctuates only in a small range, there must be some restrictions at the rhodopsin-arrestin interface. First we calculated the root mean square fluctuation (RMSF) of rhodopsin alone, arrestin alone and the complex respectively. Then we calculated the RMSF change between the RMSFs of rhodopsin and arrestin in the complex and that of the corresponding standalone proteins. Thus, positive difference values indicate that the corresponding residues become more flexible and negative values indicate the residues become more stable upon the complex formation. We plotted the value of RMSF change with residue index in Fig. 5c. From this figure we can see that the RMSF values of some residues such as F146_(rhodopsin)_, from E239_(rhodopsin)_ to T242_(rhodopsin)_ and from M76_(arrestin)_ to F80_(arrestin)_, and Y251_(arrestin)_ change significantly. We colored the complex structure according to the value of RMSF change (∆RMSF) with the blue-white-red color (Fig. 5d). The blue region at the complex interface indicate these residues become more stable upon the complex formation. Most of the blue regions are located at the complex interface, indicating that there should be some important interactions at the interface to stabilize the complex and restrict the flexibility upon the complex formation.

#### Hydrogen Bonds at the Complex Interface

**Figure 6:**
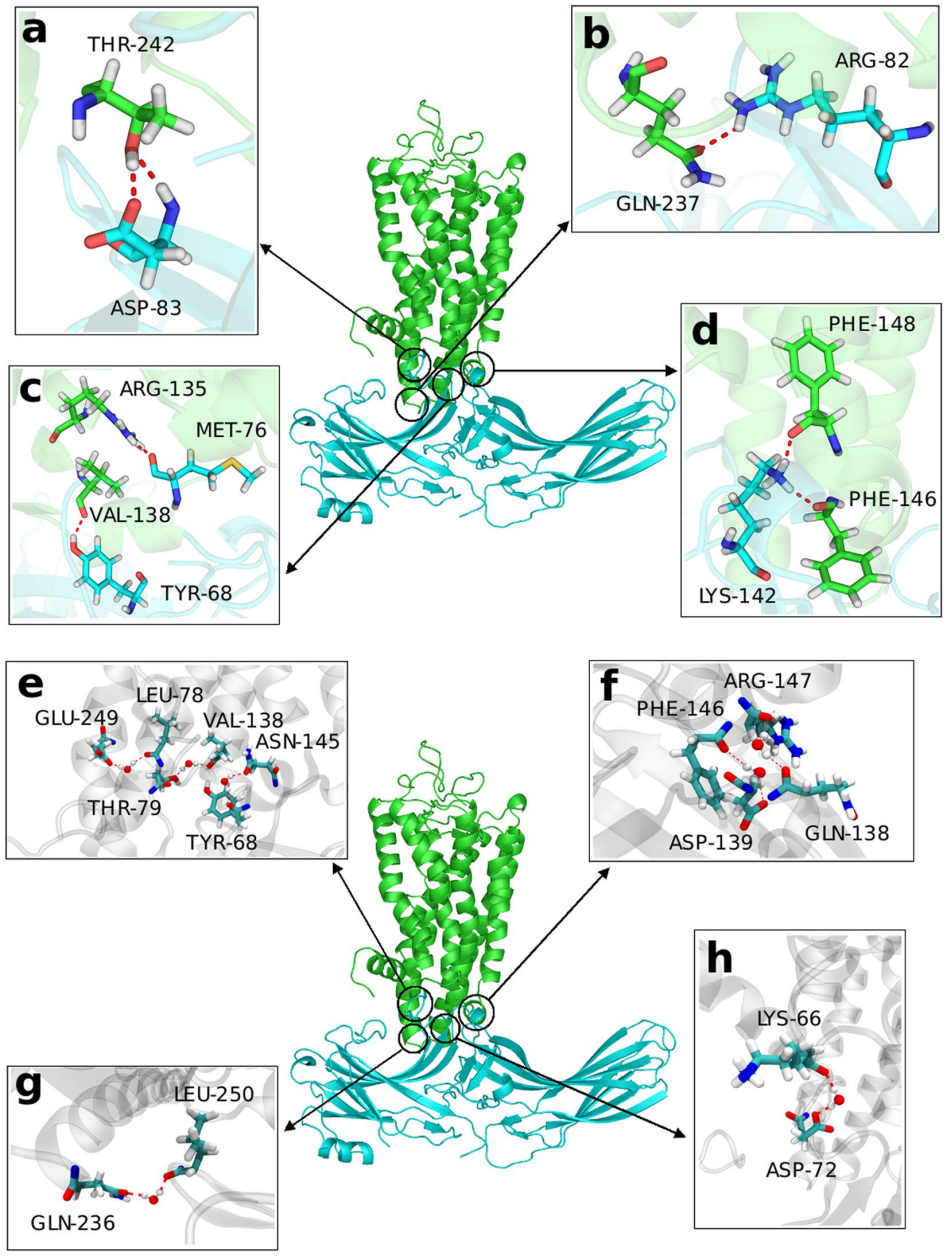
Details of the two types hydrogen bonds. Red dashed lines represent hydrogen bonds. Water was shown with the CPK model. (**a**) T242_(rhodopsin)_ side chain and D83_(arrestin)_ side chain; T242_(rhodopsin)_ side chain and D83_(arrestin)_ main chain. (**b**) Q237_(rhodopsin)_ side chain and R82_(arrestin)_ side chain. (**c**) V138_(rhodopsin)_ main chain and Y68_(arrestin)_ side chain; R135_(rhodopsin)_ side chain and M76_(arrestin)_ main chain. (**d**) F146_(rhodopsin)_ main chain and K142_(arrestin)_ side chain; F148_(rhodopsin)_ main chain and K142_(arrestin)_ side chain. (**e**) the hydrogen bonds between E249_(rhodopsin)_ and L78_(arrestin)_; the hydrogen bonds between V138_(rhodopsin)_ and T79_(arrestin)_; the hydrogen bonds between N145_(rhodopsin)_ and Y68_(arrestin)_. (**f**) the hydrogen bonds between F146_(rhodopsin)_ and D139_(arrestin)_; the hydrogen bonds between R147_(rhodopsin)_ and Q138_(arrestin)_. (**g**) the hydrogen bonds between Q236_(rhodopsin)_ and L250_(arrestin)_. (**h**) the hydrogen bonds between K66_(rhodopsin)_ and D72_(arrestin)_.

Firstly, we analyzed the hydrogen bonds (H-bonds) in the crystal structure (PDB ID: 4ZWJ) and identified four H-bonds at the complex interface, which lie between T70_(rhodopsin)_ and I73_(arrestin)_, E239_(rhodopsin)_ and R319_(arrestin)_, A233_(rhodopsin)_ and R82_(arrestin)_ and Q312_(rhodopsin)_ and I73_(arrestin)_. Subsequently, we analyzed the trajectory of rhodopsin-arrestin complex obtained by our AA MD simulation, and observed more H-bonds. For further analysis, we defined the H-bonds that lived more than 20% duration of the trajectory as long-lived H-bonds. These H-bonds are uniformly distributed at the interface, which can exist at the complex interface for a long time and are important to keep the complex steady. The entire H-bond network including the long-lived key H-bonds and transient H-bonds benefits the mutual restrictions of rhodopsin and arrestin, and thereby contribute to the stability of the complex. Some hydrogen bonds directly involve two amino acid residues in rhodopsin and arrestin directly (type A) (Fig. 6a–d), which can restrict the motion of the two proteins, especially for arrestin. We also found that water molecule can enter the complex interface. Some residues in rhodopsin and arrestin can form hydrogen bond network mediated by water molecules (type B) (Fig. 6e–h). These actually involve two hydrogen bonds: a water molecule simultaneously form two hydrogen bonds with rhodopsin and arrestin respectively. The detailed information of the two types of hydrogen bonds and their life times are shown in Table 1. Interestingly, although the H-bonds observed in the crystal structure were identified in our simulation, their occupancies are below 20% and therefore they were not included in our list of long lived H-bonds. The differences may arise from the fact that the crystallization and simulation conditions, such as temperature and buffer solution, are not completely identical.

**Table 1:** Hydrogen bonds at the rhodopsin-arrestin interface.

Residue in rhodopsin	Residue in arrestin	occupancy
Occupancy of Type A hydrogen bond		
T242 side-chain	D83	77.5%
V138	Y68	62.5%
R135	M76	51.5%
T242 main-chain	D83	44.8%
Q237	R82	40.5%
F148	K142	37.5%
F146	K142	32.2%
Occupancy of Type B Hydrogen Bond		
E249	L78	47.6%
N145	Y68	26.5%
V138	T79	24.7%
F146	D139	23.6%
K66	D72	21.9%
Q236	L250	21.6%
R147	Q138	21.0

In accordance with previous studies by Kang *et al*.^23^, the finger loop (D74, M76, G77 and L78), middle loop (Q134 and D139), and C-loop (L250 and Y251) of arrestin are the key points for rhodopsin-arrestin binding. In our MD simulations, we found that M76, L78, D139 and L250 in arrestin are able to form long-lived H-bonds with residues in rhodopsin, which makes contribution to the complex formation and stability. According to our results, T242, V138, F146 in rhodopsin can form two long-lived H-bonds, so these residues must contribute more to restrain the complex. In addition to the arrestin residues mentioned above, we also found that D83 and K142 in arrestin have strong effects on the complex binding. The study by Szczepek *et al*. shows that R135 in activated rhodopsin plays an important role upon the rhodopsin-arrestin complex formation^38^. We found that R135 in rhodopsin forms a long-lived H-bond with M76 in arrestin, which exists more than 50% of the simulation time. Our results suggest that the key residues mentioned above influence the binding and stability of rhodopsin-arrestin complex by forming long-live hydrogen bonds at the rhodopsin-arrestin interface.

#### Residue Contact at the Complex Interface

**Figure 7:**
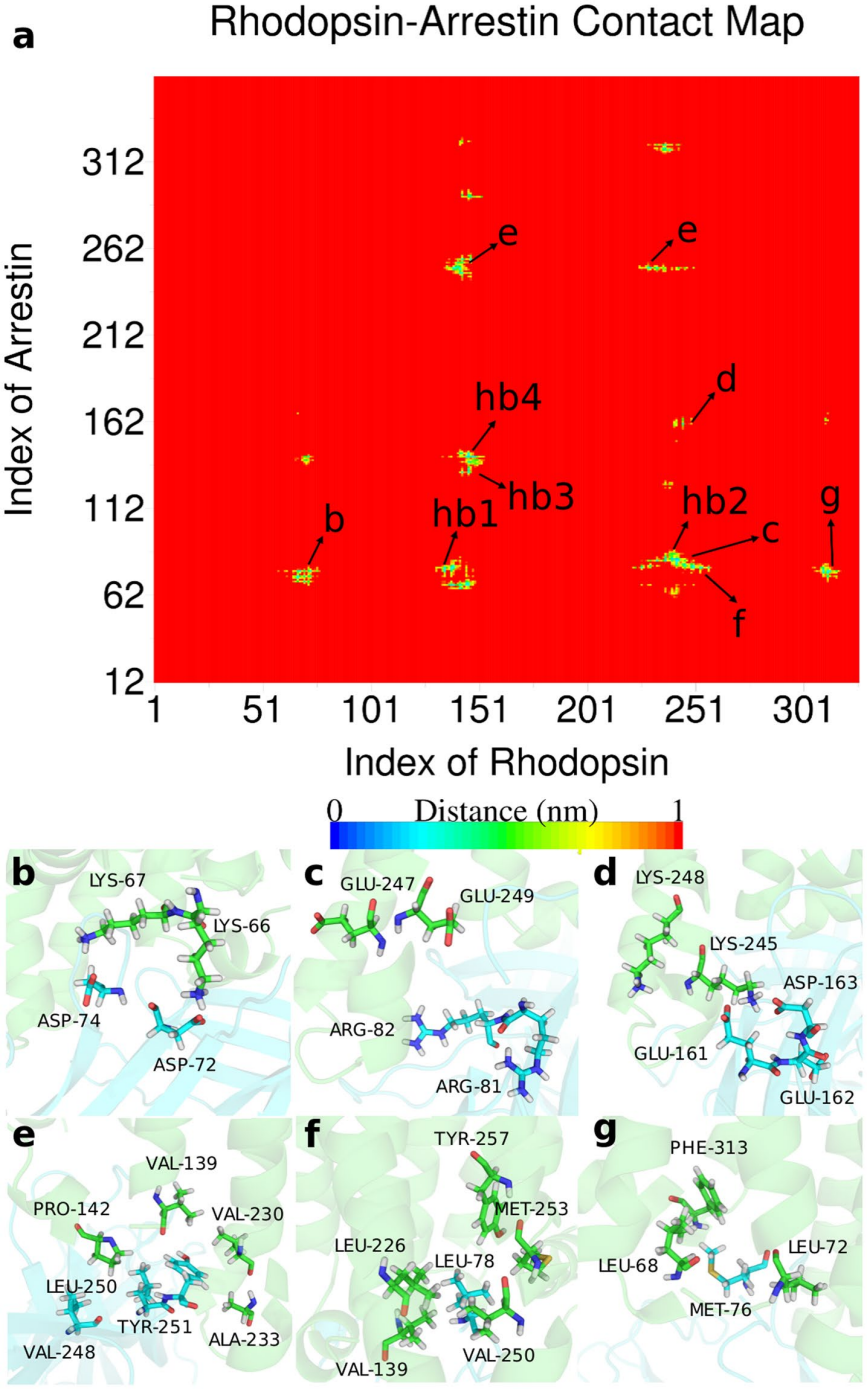
The residue contact map at the rhodopsin-arrestin interface. (**a**) Site hb1 represents type A hydrogen bonds in Fig. 6c; hb2 represents type A hydrogen bonds in Fig. 6a; hb3 represents type A hydrogen bonds in Fig. 6d; and hb4 represents type B hydrogen bonds in Fig. 6f. (**b**–**d**) Electrostatic interaction sites at the complex interface. (**e**–**g**) Hydrophobic contacts at the complex interface.

Not only the H-bonds at the complex interface, but also the electrostatic interactions and hydrophobic contacts can stabilize the rhodopsin-arrestin complex. We analyzed the residue contacts at the complex interface (Fig. 7a) and focused on the charged residues and the hydrophobic residues at the interface. We found that D72 and D74 in the finger loop of arrestin are located closely to K66 and K67 in rhodopsin (Fig. 7b); R81 and R82 in the arrestin locate closely to E247 and E249 in rhodopsin (Fig. 7c); E161, E162 and D163 in arrestin lie next to K245 and K248 in rhodopsin (Fig. 7d). As can be seen, the above contact residues in these three regions have opposite charges in rhodopsin and arrestin, so that they will attract each other and stabilize the complex. As mentioned in previous studies, D139 in arrestin is in close contact with residue F148 to N151 in rhodopsin^23^. Our results indicate that D139 has electrostatic interaction with R147 and H152 in rhodopsin. We were also interested in the hydrophobic residue contacts at the interface. According to the residue contact map (Fig. 7a), we determined three hydrophobic residue contact regions, which are shown in Fig. 7e–g. From these figures we can see that the hydrophobic residues V139, P142, V230 and A233 in rhodopsin are forming a pocket-like region where hydrophobic residues L250 and Y251 in arrestin are able to insert (Fig. 7e). A similar conformation can be found in another hydrophobic region (Fig. 7f) that L78 in arrestin embeds into a hydrophobic pocket formed by V139, L226, V250, M253 and Y257 in rhodopsin. Compared to these two regions above, fewer hydrophobic contacts exist in Fig. 7g, where M76 in arrestin interacts with the pocket formed by L68, L72 and F313 in rhodopsin. This “pocket” conformation is able to establish more hydrophobic contacts between rhodopsin and arrestin, which can act like docking sites between the two structures to stabilize the complex structure.

#### Mutation Analysis

To validate our analysis and mutation suggestions, we mutated D83A, Y68A, K142A in arrestin *in silico* and performed 200 ns MD simulations using the mutated structures. The simulation settings are the same as in the AA simulations above. We analyzed the three corresponding H-bonds at the complex interface and the result shows that there were no H-bonds existing at these sites any more. We compared the Cα-Cα distance between T242 and D83A, V138 and Y68A, F146 and K142A in the mutants and the wild type trajectories (Supplementary Fig. S4). It is clear that the Cα-Cα distances in mutant became significantly longer than in the wild type, which indicate that the arrestin tended to separate from the rhodopsin when these key H-bonds were broken. Unfortunately, due to the limitation of simulation time scale, we were not able to investigate the whole separation process. Nonetheless, the existing MD data clearly suggested that the distances between these residues significantly increased because of lacking of the three key H-bonds, indicating there importance in stabilizing the complex structure.

## Conclusion

In this study, we conducted molecular dynamics simulations for a high-resolution crystal structure of rhodopsin-arrestin complex with coarse-grained and all-atom models. Orientation analysis was performed to quantitatively study the orientations of the rhodopsin-arrestin complex, the orientation of a standalone rhodopsin, and the orientation of a standalone arrestin in or near a POPC bilayer in water. Our results show that the complex formation has a significant influence on the orientation of arrestin in a bilayer but only a minor effect on rhodopsin. Arrestin is highly restrained by the complex formation with significantly reduced fluctuation range of the orientation. The internal conformations of both rhodopsin and arrestin are changed upon the complex formation. Specifically, the TM5 and TM6 of rhodopsin move outward to make a cavity space for the middle finger loop of arrestin to move upward and stick into. Detailed analysis at the interaction interface of the complex was also made based on our MD simulation trajectories, which indicate that there exists an extensive hydrogen bond network at the interface. Among these, we identified the long lived hydrogen bonds, which are important for the stability of the complex. We also identified the key residues in rhodopsin and arrestin, which show short range electrostatic interactions and close hydrophobic contacts at the complex interface. All these identified residues are important for the rhodopsin and arrestin interactions and the complex formation.

## Electronic supplementary material

Supplementary Information
