# Supplementary material for: The orientation and stability of the GPCR-Arrestin complex in a lipid bilayer
Source: Sci Rep. 2017 Dec 5;7:16985. doi: 10.1038/s41598-017-17243-y (PMC5716996; doi:10.1038/s41598-017-17243-y)
Supplement: Supplementary file 1 — Supplementary Information [file 41598_2017_17243_MOESM1_ESM.pdf]

The orientation and stability of the GPCR-Arrestin complex in a lipid bilayer

Dali Wang[1,2], Hua Yu[2,3], Xiangdong Liu[1], Jianqiang Liu[1], Chen Song[*,2,3]

[1] *School of Physics, Shandong University, Jinan 250100, China*
[2] *Center for Quantitative Biology, Academy for Advanced Interdisciplinary Studies, Peking University, Beijing 100871, China*
[3] *Peking-Tsinghua Center for Life Sciences, Academy for Advanced Interdisciplinary Studies, Peking University, Beijing 100871, China*
** Correspondence: c.song@pku.edu.cn*

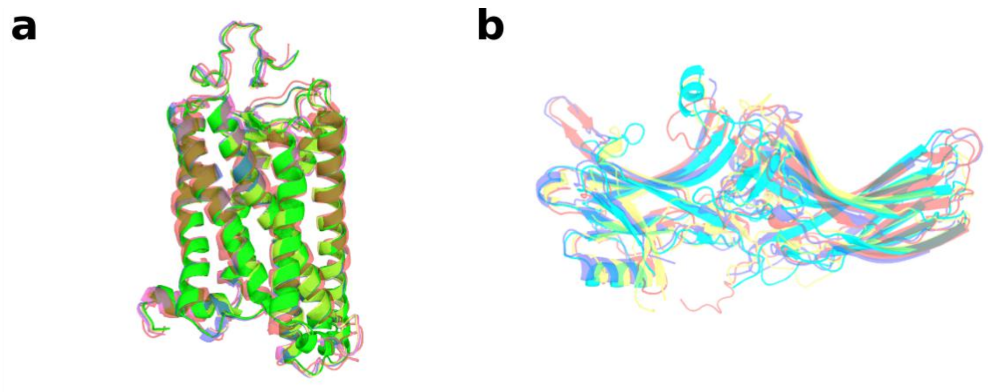

Supplementary Figure S1: Overlaid structures of the standalone models. (a) The structure extracted from the complex (PDB ID 4ZWJ) was colored in green, from 2x72 (PDB ID) colored in blue, 4a4m colored in yellow, 5dys colored in magenta and 2j4y colored in red. (b) The structure extracted from the complex was colored in cyan, 1g4m colored in yellow, 3ugu colored in blue, and 1ayr colored in red. As can be seen, the overall structures are well overlaid, indicating there are no major global conformational changes upon the complex formation. Local conformational differences do exit, mainly at the binding interface and loop regions.

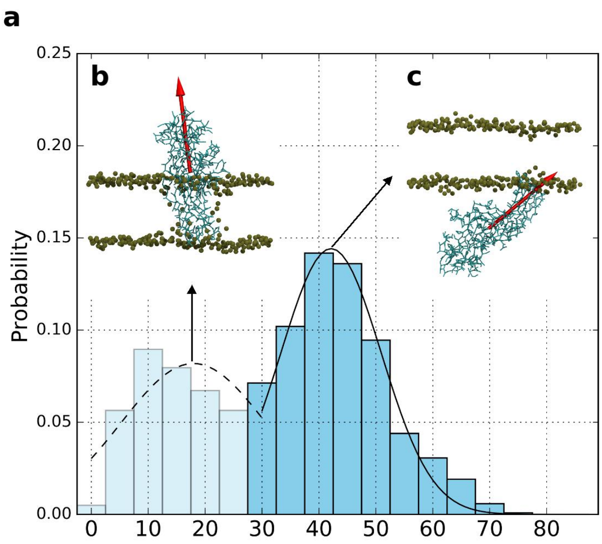

Supplementary Figure S2: The orientations of the standalone arrestin around a POPC bilayer. (a) The standalone arrestin orientation distribution. The transparent bars indicate unrealistic orientation of arrestin due to misassembly, and the opaque bars indicate the probable orientations. (b) An example of the misassembled system, in which the arrestin lies embedded in a lipid bilayer and its first principal axis is almost parallel to the z-axis. This was due to misassembly and therefore discarded for further analysis. (c) The most probable orientation of the standalone arrestin on a POPC bilayer.

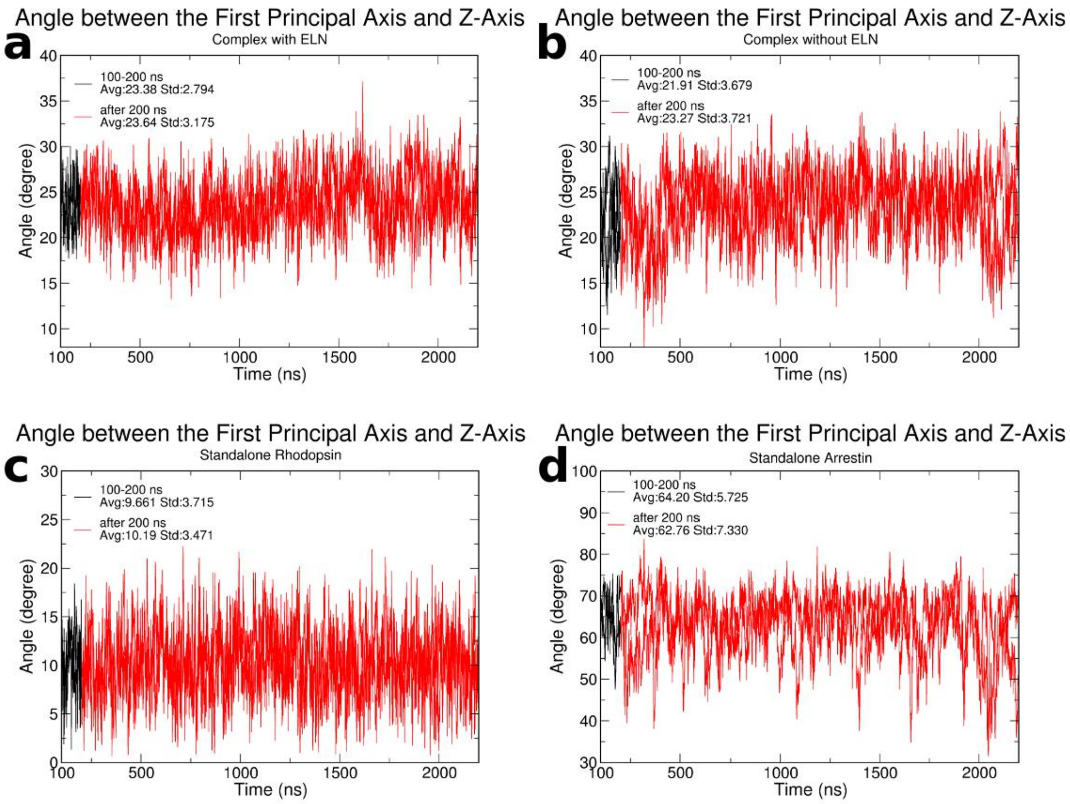

Supplementary Figure S3: The orientation evolution of proteins in the extended trajectories. (a)-(d) The orientation evolution of the complex, the complex without ELN, the standalone rhodopsin and the standalone arrestin in the 100-2200 ns trajectories. The first 100-ns trajectories were considered as equilibration and discarded for analysis.

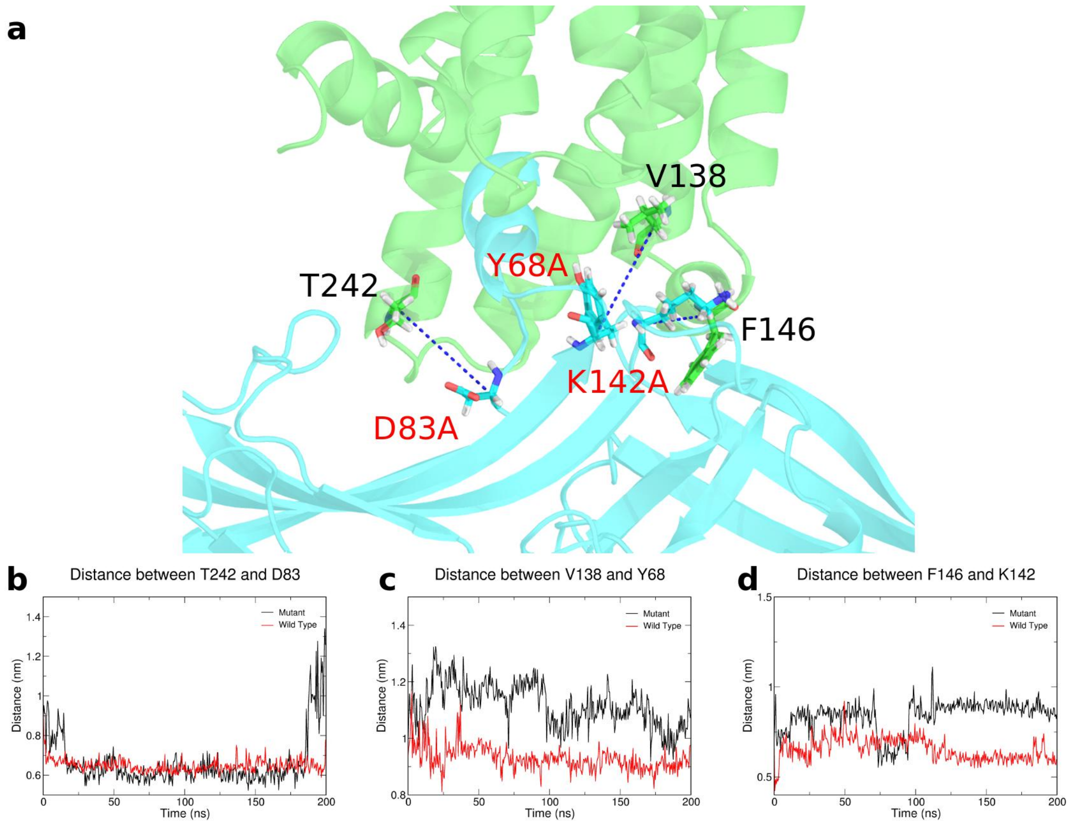

Supplementary Figure S4: Distance between the three pairs of residues that form the key H-bonds stabilizing the complex. (a) The three pairs of residues in the complex structure. (b)-(d) Cα-Cα distance between T242$_{\text{(rhodopsin)}}$ and D83A$_{\text{(arrestin)}}$, V138$_{\text{(rhodopsin)}}$ and Y68A$_{\text{(arrestin)}}$, and F146$_{\text{(rhodopsin)}}$ and K142A$_{\text{(arrestin)}}$ before and after mutation in our simulations. As can be seen, after mutation, the distances became significantly larger, indicating the complex became less compact and stable.

Supplementary Table

Supplementary Table S1

RMSD of rhodopsin

PDB ID	Sequence Identity (%)	Resolution (Å)	RMSD (Å)
2x72	93.23	3.0	1.183
4a4m	93.25	3.3	1.190
5dys	92.64	2.3	1.255
2j4y	92.24	3.4	2.611

RMSD of arrestin

PDB ID	Sequence Identity (%)	Resolution (Å)	RMSD (Å)
1g4m	62.21	1.9	2.998
3ugu	87.32	1.8	3.501
1ayr	92.64	3.3	3.985

Table Legend

RMSDs between the initial standalone structures in our MD simulations (extracted from the complex structure with PDB ID 4ZWJ) and other standalone X-ray structures with high sequence identity. The structures were aligned based on C-alpha atoms and RMSD calculations were performed for C-alpha atoms. The values are small for rhodopsin and mild for arrestin, indicating there are no major global conformational changes occurred upon the complex formation. The conformational changes mainly occurred at the binding interface and the loop regions of arrestin. (1) RMSDs of rhodopsin. (2) RMSDs of arrestin.
